# Supplementary material for: Aluminum depletion induced by co-segregation of carbon and boron in a bcc-iron grain boundary
Source: Nat Commun. 2021 Oct 14;12:6008. doi: 10.1038/s41467-021-26197-9 (PMC8516984; doi:10.1038/s41467-021-26197-9)
Supplement: Supplementary file 1 — Supplementary Information [file 41467_2021_26197_MOESM1_ESM.pdf]

Supplementary Information for Aluminum depletion induced by co-segregation of carbon and boron in a bcc-iron grain boundary

Ahmadian, A.[1,*], Scheiber, D.[2], Zhou, X.[1], Gault, B.[1], Liebscher, C. H.[1], Romaner, L.[2], and Dehm, G.[1]

[1]*Max-Planck-Institut fuer Eisenforschung GmbH, 40237 Düsseldorf, Germany*
[2]*Materials Center Leoben GmbH, 8700 Leoben, Austria*
[*]*Corresponding author: a.ahmadian@mpie.de*

Supplementary Tables

Solute	D_0 [m/s^2]	E_a [eV]	Ref.
Al	1.80×10^{-4}	2.364	[1]
C	3.94×10^{-7}	0.831	[1]
B	3.19×10^{-7}	2.310	[2]

Supplementary Table 1: Diffusion data employed for the segregation kinetics simulations.

Element	Al	C	B	O	P	S	Si
Content (at.%)	4	0.05	0.001	0.22	< 0.003	0.001	0.005

Supplementary Table 2: Wet chemical analysis of the Fe bicrystal showing the bulk concentrations.

Supplementary Figures

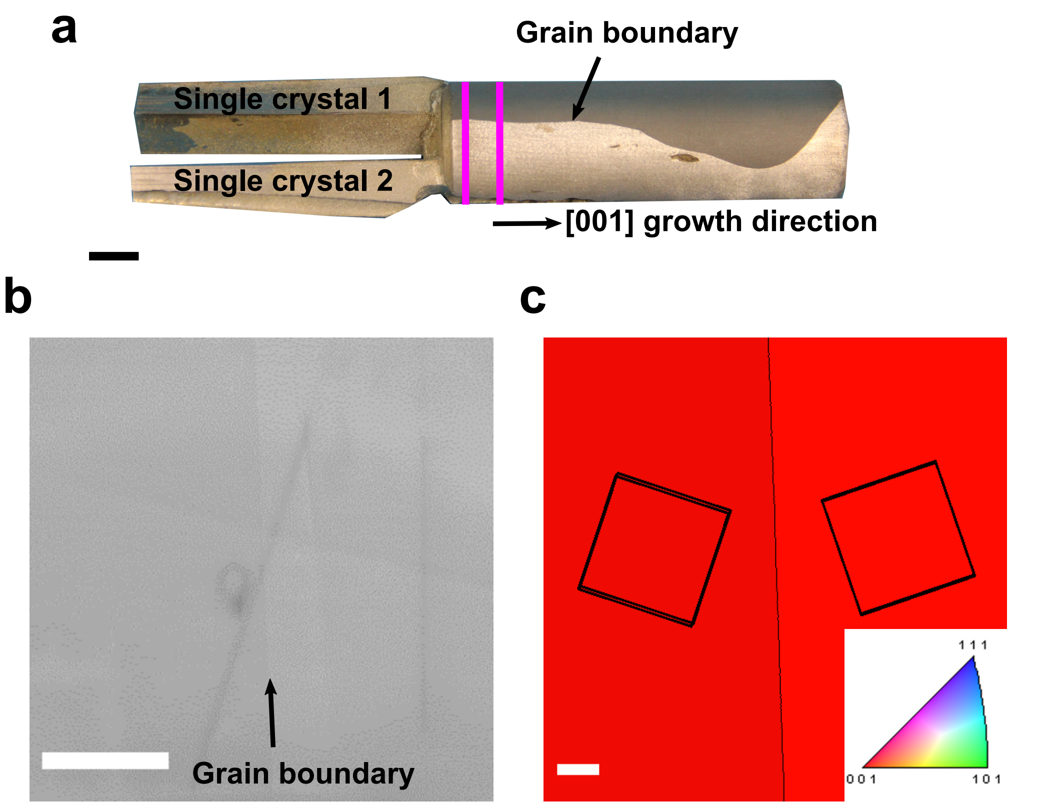

Supplementary Figure 1: Global characterization of the GB. a Side view image of the bicrystal including the single crystal ingots to fabricate it. The growth direction is [0 0 1]. Samples were investigated from the initial part (labelled by magenta lines), where the GB started to grew. **b** SEM image of the bicrystal top surface (along the [0 0 1] tilt axis) after final polishing. The GB is indicated by the black arrow. The GB runs straight and shows no steps or curvatures at the micron scale. **c** EBSD scan of the bicrystal shows a clear [0 0 1] texture of both grains. In each grain the orientation of the unit cells is shown by the black rectangles indicating a symmetric misorientation of 37.5°. Further analsis of the polefigure the GB plane was obtained to be (3 1 0) into both grains. The Scale bar in **a** is 10 mm in **b** 5 μm and in **c** 1 μm.

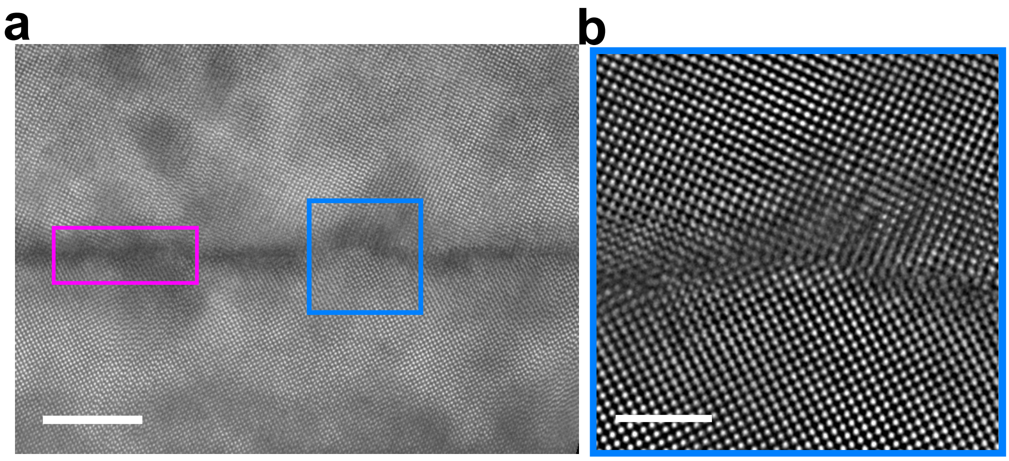

Supplementary Figure 2: High resolution imaging of distorted GB structure. **a** HAADF-STEM image of the GB showing small steps (magenta rectangle), where the boundary plane shows facetting. Besides the small steps, large distortions are highlighted in the blue box and a higher magnified image is shown in **b**. The large step caused a large amount of strain - especially onto the upper grain. Thereby, the kite-structure is not visible anymore. The Scale bar in **a** is 5 nm and in **b** 2 nm.

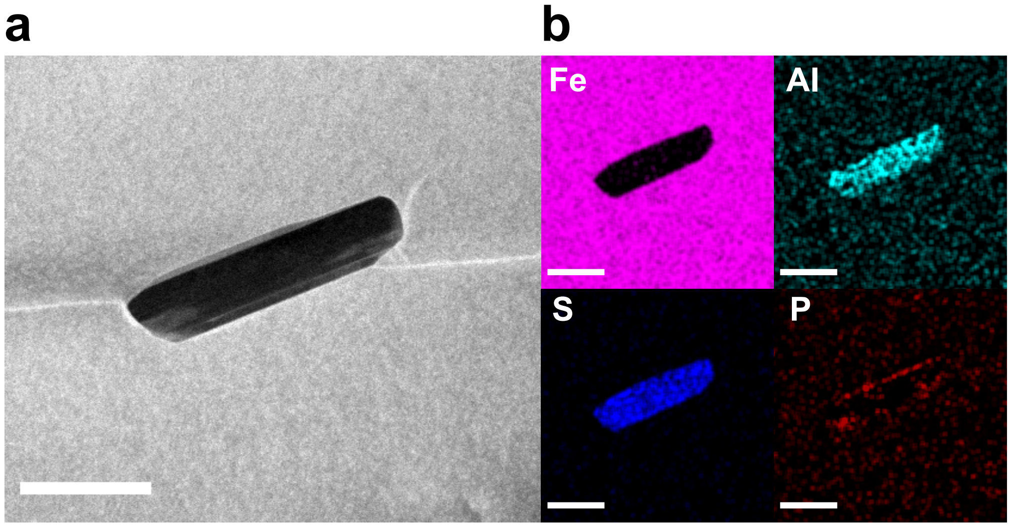

Supplementary Figure 3: Formation of precipitates at the GB. **a** HAADF-STEM image of the GB intersecting with a large precipitate. **b** The corresponding EDS elemental maps for Fe, Al, S and P. The Scale bar in **a** and **b** is 100 nm.

Supplementary References

[1] Mehrer, H. & Stolica, N. *Diffusion in solid metals and alloys.* vol. 26 (Springer-Verlag, Berlin/Heidelberg, 1990).

[2] Fors, D. H. R. & Wahnström, G. Nature of boron solution and diffusion in α-Iron. *Phys. Rev. B* **77**, 132102 (2008).
